# Comparison of hydroxychloroquine, lopinavir/ritonavir, and standard of care in critically ill patients with SARS-CoV-2 pneumonia: an opportunistic retrospective analysis

**DOI:** 10.1186/s13054-020-03117-9

**Published:** 2020-07-11

**Authors:** Marie Lecronier, Alexandra Beurton, Sonia Burrel, Luc Haudebourg, Robin Deleris, Julien Le Marec, Sara Virolle, Safaa Nemlaghi, Côme Bureau, Pierre Mora, Martin De Sarcus, Olivier Clovet, Baptiste Duceau, Paul Henri Grisot, Marie Hélène Pari, Jérémy Arzoine, Ulrich Clarac, David Boutolleau, Mathieu Raux, Julie Delemazure, Morgane Faure, Maxens Decavele, Elise Morawiec, Julien Mayaux, Alexandre Demoule, Martin Dres

**Affiliations:** 1 AP-HP. Sorbonne Université, Hôpital Pitié-Salpêtrière, Service de Pneumologie, Médecine intensive – Réanimation (Département “R3S”), Paris, France; 2 Sorbonne Université, INSERM, UMR_S 1158 Neurophysiologie respiratoire expérimentale et clinique, Paris, France; 3 Sorbonne Université, INSERM UMR S 1136, Institut Pierre Louis d’Epidémiologie et de Santé Publique, Team 3 THERAVIR, Paris, France; 4 AP-HP. Sorbonne Université, Hôpital Pitié-Salpêtrière, Service de Virologie, Centre National de Référence Herpès virus, Paris, France; 5 AP-HP. Sorbonne Université, Hôpital Pitié-Salpêtrière, Département d’anesthésie réanimation, Paris, France

**Keywords:** SARS-CoV-2, Intensive care unit, Hydroxychloroquine, Lopinavir/ritonavir, Standard of care

## Abstract

**Background:**

The severe acute respiratory syndrome coronavirus-2 (SARS-CoV-2) outbreak is spreading worldwide. To date, no specific treatment has convincingly demonstrated its efficacy. Hydroxychloroquine and lopinavir/ritonavir have potential interest, but virological and clinical data are scarce, especially in critically ill patients.

**Methods:**

The present report took the opportunity of compassionate use and successive drug shortages to compare the effects of two therapeutic options, lopinavir/ritonavir and hydroxychloroquine, as compared to standard of care only. The primary outcomes were treatment escalation (intubation, extra-corporeal membrane oxygenation support, or renal replacement therapy) after day 1 until day 28. Secondary outcomes included ventilator-free days at day 28, mortality at day 14 and day 28, treatment safety issues and changes in respiratory tracts, and plasma viral load (as estimated by cycle threshold value) between admission and day 7.

**Results:**

Eighty patients were treated during a 4-week period and included in the analysis: 22 (28%) received standard of care only, 20 (25%) patients received lopinavir/ritonavir associated to standard of care, and 38 (47%) patients received hydroxychloroquine and standard of care. Baseline characteristics were well balanced between the 3 groups. Treatment escalation occurred in 9 (41%), 10 (50%), and 15 (39%) patients who received standard of care only, standard of care and lopinavir/ritonavir, and standard of care and hydroxychloroquine, respectively (*p* = 0.567). There was no significant difference between groups regarding the number of ventilator-free days at day 28 and mortality at day 14 and day 28. Finally, there was no significant change between groups in viral respiratory or plasma load between admission and day 7.

**Conclusion:**

In critically ill patients admitted for SARS-CoV-2-related pneumonia, no difference was found between hydroxychloroquine or lopinavir/ritonavir as compared to standard of care only on the proportion of patients who needed treatment escalation at day 28. Further randomized controlled trials are required to demonstrate whether these drugs may be useful in this context.

## Background

Severe acute respiratory syndrome coronavirus-2 (SARS-CoV-2) outbreak is a major epidemic threat that is spreading worldwide since January 2020 [1]. About one-third of patients with SARS-CoV-2 pneumonia hospitalized for acute respiratory failure will require admission to the intensive care unit (ICU), where mortality is high [2]. Up to date, no specific treatment has convincingly demonstrated its efficacy in this setting [3], pointing out the need for observational data that would help designing future randomized trials. Chloroquine is a widespread anti-malarial drug that is also associated with immunomodulatory effects [4, 5]. Hydroxychloroquine is an analog of chloroquine, widely used in the management of some diseases like systemic lupus, for which there are fewer concerns about clinical tolerance. Hydroxychloroquine has an in vitro anti-SARS-CoV-2 activity [6] and reduces viral load in asymptomatic patients and in those with a mild form of illness, without any benefit on clinical outcome [7, 8]. Likewise, lopinavir/ritonavir is a combination of protease inhibitor used in human immunodeficiency virus infection that has gained interest in the context of SARS-CoV-2 outbreak due to its in vitro inhibitory activity against SARS-CoV-1 [9]. A recent open-label controlled randomized trial failed to demonstrate any benefit of lopinavir/ritonavir beyond standard care [10], but the potential interest of the treatment has not been ruled out in the specific setting of the most seriously ill patients. To date, no study has reported the potential impact of these antiviral therapies in critically ill patients despite the high mortality observed in this population [11, 12].

The present report took the opportunity of successive drug shortages and compassionate uses initiated before the start of randomized controlled trials. This observational report that is not a randomized controlled trial reflects the local experience of a national expert center since the beginning of the SARS-CoV-2 outbreak to compare the effects of lopinavir/ritonavir, hydroxychloroquine, and standard of care only on clinical outcomes and viral load reduction in patients with a severe SARS-CoV-2 pneumonia requiring ICU admission.

## Patients and methods

Guidelines for reporting this retrospective study were from the Strengthening the Reporting of Observational Studies in Epidemiology (STROBE) Statement [13]. This was a retrospective analysis, conducted over 1 month, from March 4, 2020, to April 6, 2020, in a medical intensive care unit (32 beds) before that any randomized controlled trial on SARS-CoV-2 had started. The Research Ethics Committee of Sorbonne University approved the project (CER 2020-36). Oral information about this retrospective analysis was given to patients or relatives.

### Study population

Since our hospital is a national reference center for emerging biological risks, only patients with suspected or confirmed SARS-CoV-2 infection were admitted in our medical ICU during this period as long as they presented clinical signs of severity. The data from all prospective patients admitted to our medical ICU and who fulfilled the following criteria were studied: (1) acute respiratory failure as defined by severe hypoxemia requiring either a high level of oxygen via facemask (> 6 L/min to achieve SpO2 > 90%), high flow oxygen therapy (with a minimum of 30 L/min and 50% FiO2 to achieve SpO2 > 90%), or invasive mechanical ventilation and (2) proven infection by SARS-CoV-2 defined by positive reverse transcriptase polymerase chain reaction (RT-PCR) assay targeting the E (envelope) gene of SARS-CoV-2, obtained from nasopharyngeal swab or lower respiratory tracts [14]. Patients who received anti-viral treatments other than lopinavir/ritonavir or hydroxychloroquine or who received both of these treatments were not analyzed.

### Treatments

Somewhat by accident, three therapeutic approaches were consecutively implemented. During the first period, each new patient admitted in our ICU was receiving, in addition to standard of care, lopinavir/ritonavir (400 mg twice daily, oral route down the nasogastric tube in syrup form) for 5 days as per our local disease control policies. During the second period, due to a lopinavir/ritonavir shortage, the local policy was changed to hydroxychloroquine (200 mg, twice a day, oral route) for each new patient admitted in our ICU. During the last period, no specific treatment was given besides the standard of care. Soon after, patients started to be included in randomized controlled trials. Across each period, patients received standard of care that consisted of ventilatory support, antibiotic agents whenever needed, vasopressors, renal replacement therapy, and ECMO.

### Data collection

The following data were extracted from each patient’s electronic medical chart: age, gender, clinical and biological variables upon admission, and time between symptoms’ onset and ICU admission. Simplified Acute Physiology Score (SAPS) 2 and Sequential Organ Failure Assessment (SOFA) were calculated upon ICU admission. Advanced life support measures taken during the ICU stay such as invasive mechanical ventilation, ECMO, vasopressor support, and renal replacement therapy were also collected. Finally, we recorded the length of ICU stay, time spent under invasive mechanical ventilation, and biological variables. Mortality was assessed at day 14 and day 28 after ICU admission. SARS-CoV-2 load in respiratory tracts (nasopharyngeal swab or tracheal aspiration) and plasma SARS-CoV-2 load were collected within 24 h of ICU admission and at day 7. The cycle threshold (CT) value of RT-PCR was used as an indicator of the viral load in clinical samples, the lower the CT value, and the higher the viral load. PCR was considered negative when CT was > 45.

### Outcomes analyzed

The primary outcome was treatment escalation occurring after day 1 after ICU admission until day 28. Treatment escalation was defined by the initiation of at least one life support intervention among intubation, ECMO, or renal replacement therapy. Secondary clinical outcomes included ventilator-free days at day 28 (zero ventilator-free day was attributed to a patient who died) and mortality on day 14 and day 28. Last, secondary virological outcomes were viral load changes in respiratory tracts and plasma between admission and day 7.

### Statistical analysis

Continuous variables are expressed as median (25–75, interquartile range, IQR) and categorical variables are expressed as number and relative frequencies (%). Patients were categorized a posteriori into the three groups according to the treatment that they received. Continuous variables were tested for normality using the Kolmogorov-Smirnov normality test. Gaussian variables were compared using an ordinary ANOVA test and non-normally distributed variables using a Kruskal-Wallis test. Categorical variables were compared with chi-square test or Fisher’s exact test. Kaplan-Meier curves were computed for the proportion of patients who needed treatment escalation and were compared using log-rank test. Due to the retrospective nature of the study, no sample size calculation was performed. A convenience sample of patients corresponding to the number of patients admitted in the ICU during the first 4 weeks of the outbreak was deemed appropriate. For final comparisons, a two-tailed *p* value less than or equal to 0.05 was considered statistically significant. The statistical analysis was performed by using Prism 8.3.0 software (GraphPad Software, USA).

## Results

**Fig. 1:**
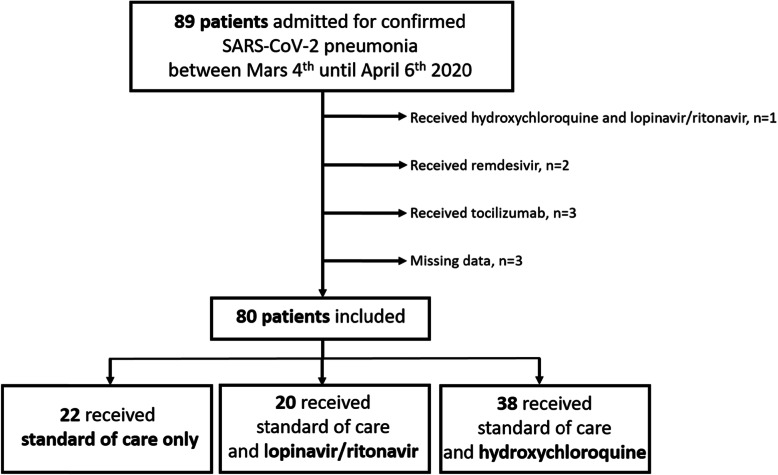
Flow chart of the study

During the study period, 89 patients were admitted to the ICU and 80 patients were included in the analysis (see the flow chart in Fig. 1). Twenty-two patients (28%) received standard of care only, 20 (25%) patients received standard of care and lopinavir/ritonavir, and 38 (47%) patients received standard of care and hydroxychloroquine. Patients were admitted to the ICU with a median of 8 (6–11) days after the onset of symptoms, a duration that was similar across three groups (Table 1). Baseline characteristics were not different across the three groups, in particular regarding their age, severity scores (SAPS2 and SOFA), and the presence of comorbidities (Table 1). Respiratory and organ support upon admission were not different between the three groups. During the first period, patients received lopinavir/ritonavir for 4 (IQR, 2–5) days and during the second period, they received hydroxychloroquine for 5 (IQR, 3–7) days.

**Table 1:** Patient’s characteristics upon intensive care unit admission (first 24 h)

	Total ***n*** = 80	Standard of care ***n*** = 22	Lopinavir/ritonavir ***n*** = 20	Hydroxychloroquine ***n*** = 38	***p*** value
Age, year	57 (53–68)	63 (54–70)	55 (49–61)	59 (53–66)	0.109
Male gender, *n* (%)	64 (80)	18 (82)	15 (75)	31 (82)	0.812
BMI, kg/m^2^	29 (26–32)	28 (26–31)	30 (28–33)	27 (25–32)	0.261
SAPS2	33 (25–40)	32 (27–39)	33 (29–41)	33 (24–40)	0.758
SOFA	7 (4–9)	7 (4–10)	7 (3–10)	7 (4–8)	0.797
Symptoms duration prior ICU, days	8 (6–11)	9 (7–14)	8 (6–10)	9 (7–12)	0.100
**Comorbidities**					
Active smoking, *n* (%)	4 (5)	2 (9)	1 (5)	1 (3)	0.542
Chronic hypertension, *n* (%)	30 (38)	9 (41)	11 (55)	10 (26)	0.093
Diabetes mellitus, *n* (%)	21 (26)	6 (27)	6 (30)	9 (24)	0.866
Chronic respiratory disease, *n* (%)	10 (13)	6 (27)	2 (10)	2 (5)	0.042
Chronic cardiac disease, *n* (%)	16 (20)	6 (27)	2 (10)	8 (21)	0.367
Immunosuppression, *n* (%)	9 (11)	2 (9)	2 (10)	5 (13)	0.873
**Clinical variables**					
Temperature ≥ 38.5 °C, *n* (%)	20 (25)	3 (14)	6 (30)	11 (29)	0.677
Respiratory rate, min^−1^	30 (24–35)	30 (27–38)	25 (20–30)	31 (25–37)	0.008
Mean arterial blood pressure, mmHg	69 (59–82)	61 (55–74)	65 (60–82)	73 (61–83)	0.072
**Biological variables**					
Lymphocyte count, 10^−9^/l	0.82 (0.63–1.19)	0.82 (0.56–0.99)	1.12 (0.67–1.41)	0.76 (0.58–1.17)	0.261
Creatinine, μmol l^−1^	87 (67–118)	91 (72–138)	78 (66–108)	86 (62–109)	0.387
PaO_2_/FiO_2_, mmHg	130 (101–178)	134 (101–172)	172 (112–230)	127 (100–159)	0.221
**Respiratory support within 24 h**					
Invasive mechanical ventilation, *n* (%)	56 (70)	15 (68)	16 (80)	25 (66)	0.520
High flow nasal oxygenation, *n* (%)	9 (11)	4 (18)	0 (0)	5 (13)	0.155
Oxygen mask, *n* (%)	15 (19)	3 (14)	4 (20)	8 (21)	0.767
**Organ support within 24 h**					
Vasopressors, *n* (%)	44 (55)	13 (59)	12 (60)	19 (50)	0.693
Renal replacement therapy, *n* (%)	1 (3)	0 (0)	0 (0)	1 (3)	0.640

Categorical variables are expressed as absolute value (%) and continuous variables as median (IQR). *p* values are given for the comparison between groups of treatment

*IQR*, interquartile range, *BMI* body mass index, *SAPS2* Simplified Acute Physiology Score, *SOFA* Sepsis-Related Organ Failure Assessment, *ICU* intensive care unit

### Primary outcomes

**Fig. 2:**
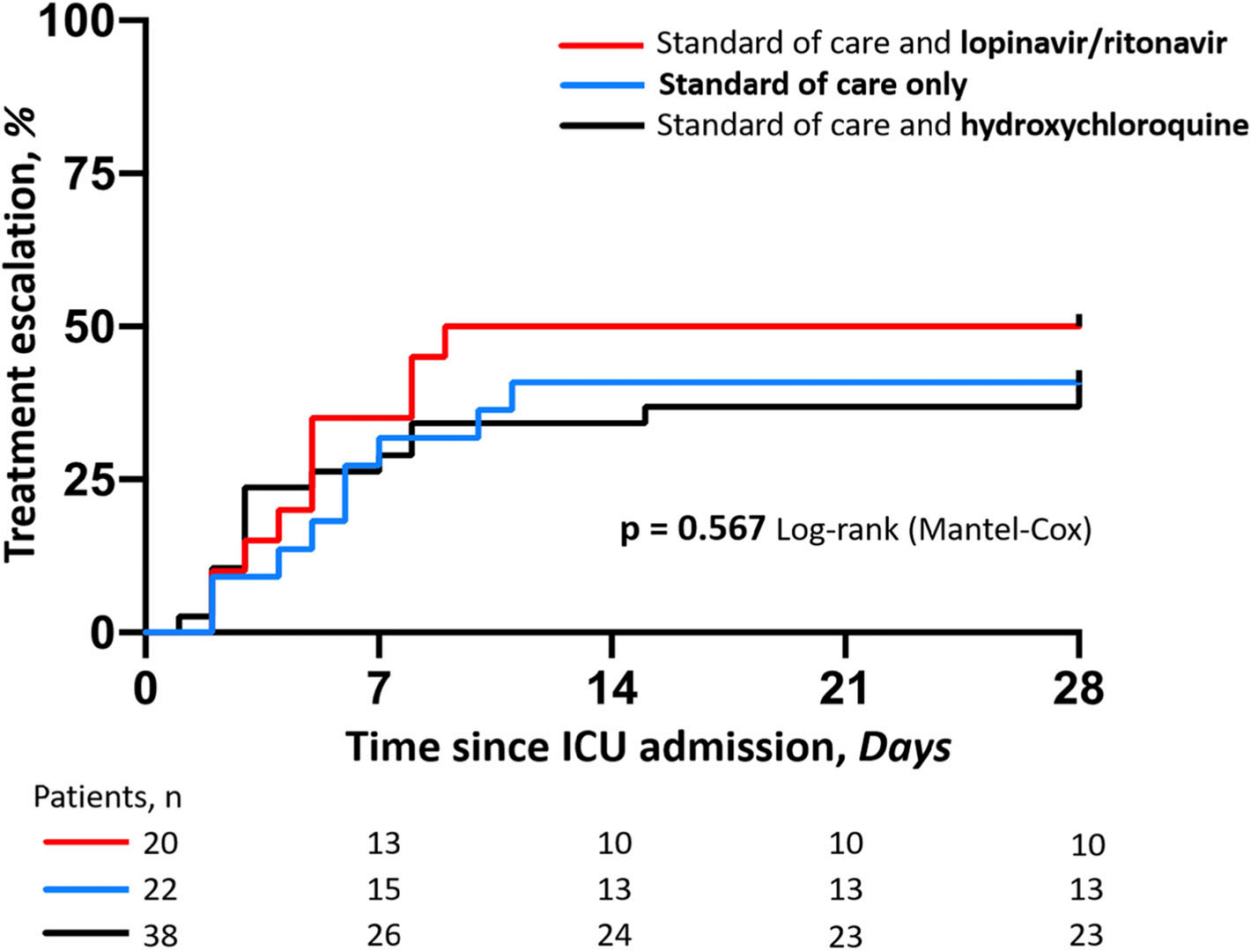
Primary endpoint. Kaplan-Meier curve with the proportion of patients who needed treatment escalation (intubation, extracorporeal membrane oxygenation support, and renal replacement therapy) during the first 28 days of the intensive care unit stay. ICU, intensive care unit

The number of patients who needed treatment escalation after day 1 until day 28 of the intensive care unit stay was 9 (41%), 10 (50%), and 15 (39%) for those who received standard of care only, standard of care and lopinavir/ritonavir, and standard of care and hydroxychloroquine, respectively (Table 2). There was no difference between the 3 groups (*p* = 0.567) (Fig. 2).

**Table 2:** Primary and secondary outcomes

Outcomes	Total ***n*** = 80	Standard of care ***n*** = 22	Lopinavir/ritonavir ***n*** = 20	Hydroxychloroquine ***n*** = 38	***p*** value
Treatment escalation after day 1 until day 28, *n* (%)	34 (43)	9 (41)	10 (50)	15 (39)	0.731
Intubation, *n* (%)	8 (10)	2 (9)	1 (5)	5 (13)	0.607
ECMO, *n* (%)	13 (16)	4 (18)	4 (20)	5 (13)	0.766
Renal replacement therapy, *n* (%)	19 (24)	4 (18)	8 (40)	7 (18)	0.143
Time between ICU admission and treatment escalation, days	5 (3–7)	6 (4–9)	5 (3–8)	3 (2–7)	0.441
Ventilator-free days at day 28	7 (0–22)	0 (0–23)	9 (0–16)	9 (0–23)	0.546
14-day mortality, *n* (%)	22 (28)	9 (41)	5 (25)	8 (21)	0.242
28-day mortality, *n* (%)	25 (31)	9 (41)	7 (35)	9 (24)	0.350

Categorical variables are expressed as absolute value (%) and continuous variables as median (IQR). *p* values are given for the comparison between groups of treatment

*IQR* interquartile range, *ECMO* extracorporeal membrane oxygenation, *ICU* intensive care unit

### Clinical secondary outcomes

Clinical secondary outcomes are presented in Table 2. There was no difference across groups regarding the number of ventilator-free days and mortality on day 14 and day 28. No significant differences were observed regarding safety and tolerance of lopinavir/ritonavir and hydroxychloroquine (Table 3).

**Table 3:** Safety and tolerance within the first 7 days following treatment initiation

Tolerance issues	Total ***n*** = 80	Standard of care ***n*** = 22	Lopinavir/ritonavir ***n*** = 20	Hydroxy-chloroquine ***n*** = 38	***p*** value
Neutropenia, *n* (%)	1 (1)	0 (0)	1 (5)	0 (0)	0.219
Anemia, *n* (%)	7 (9)	2 (9)	2 (10)	3 (8)	0.962
Thrombocytopenia, *n* (%)	10 (13)	2 (9)	3 (15)	5 (13)	0.834
Increased aspartate aminotransferase > 5 N, *n* (%)	20 (25)	6 (27)	7 (35)	7 (18)	0.367
Increased alanine aminotransferase > 5 N, *n* (%)	14 (18)	3 (14)	3 (15)	8 (21)	0.724
Acute renal failure KDIGO ≥ 2, *n* (%)	40 (50)	12 (55)	12 (60)	16 (42)	0.381
Prolonged QT interval*, *n* (%)	1 (1)	0 (0)	0 (0)	1 (3)	0.571

Categorical variables are expressed as absolute value (%). *p* values are given for the comparison between groups of treatment

*KDIGO* Kidney disease improving global outcome

*QT interval is prolonged when > 450 ms in men and > 470 ms in women

### Virological secondary outcomes

Among the 80 patients included in the analysis, 67 (84%) had a CT value available in the respiratory tracts upon admission. The main reasons for the lack of CT values were related to the patient’s sampling prior ICU admission (*n* = 11) and technical issues (*n* = 2). Concerning the plasma sample, 23 (29%) had a CT value available on admission. CT value upon admission was not significantly different between groups (*p* = 0.483) (Table 4). The proportion of patients with negative respiratory and plasma RT-PCR on day 7 was similar between groups (Table 4). Finally, changes in SARS-CoV-2 respiratory load between admission and day 7 were not different between groups: 2 (IQR, − 1–7), 10 (IQR, 5–18), and 8 (IQR, 4–13) for those who received standard of care only, standard of care and lopinavir/ritonavir, and standard of care and hydroxychloroquine, respectively (*p* = 0.128).

**Table 4:** Virological findings on admission and on day 7

	Total	Standard of care	Lopinavir/ritonavir	Hydroxy-chloroquine	***p*** value
Respiratory RT-PCR at admission					
Patients, *n*	80	22	20	38	–
Samples analyzed, *n* (%)	67 (84)	21 (95)	13 (65)	33 (87)	–
CT value	25 (23–30)	26 (24–31)	25 (23–30)	24 (21–29)	0.498
Plasma RT-PCR at admission					
Patients, *n*	80	22	20	38	–
Samples analyzed, *n* (%)	23 (29)	5 (23)	3 (15)	15 (39)	–
Negative RT-PCR, *n* (%)	11 (48)	4 (80)	0 (0)	7 (47)	0.09
Positive RT-PCR, *n* (%)	12 (52)	1 (20)	3 (15)	8 (53)	
CT value	30 (29–33)	35 (35–35)	30 (29–31)	30 (29–32)	NA*
Respiratory RT-PCR at day 7					
Patients, *n*	80	22	20	38	–
Samples analyzed, *n* (%)	51 (64)	14 (64)	11 (55)	26 (68)	–
Negative RT-PCR, *n* (%)	14 (27)	2 (14)	5 (45)	7 (27)	0.222
Positive RT-PCR, *n* (%)	37 (73)	12 (86)	6 (55)	19 (73)	
CT value	29 (27–34)	28 (27–32)	29 (25–29)	32 (28–35)	0.514
Plasma RT-PCR at day 7					
Patients, *n*	80	22	20	38	–
Samples analyzed, *n* (%)	54 (68)	13 (59)	13 (65)	28 (74)	–
Negative RT-PCR, *n* (%)	42 (78)	11 (85)	9 (69)	22 (79)	0.634
Positive RT-PCR, *n* (%)	12 (22)	2 (15)	4 (31)	6 (21)	
CT value	32 (30–36)	38 (38–38)	31 (30–32)	32 (29–34)	0.237

Categorical variables are expressed as absolute value (%) and continuous variables as median (IQR)

*IQR* interquartile range, *RT-PCR* reverse transcription polymerase chain reaction, *CT* cycle threshold

*NA because the number of samples analyzed was to low (*n* < 2)

## Discussion

The compassionate use of drugs and a supply shortage provided us with the opportunity to evaluate the association between hydroxychloroquine, lopinavir/ritonavir, or standard of care only and treatment escalation as defined by the need for intubation, ECMO and renal replacement therapy in critically ill patients presenting with SARS-CoV-2-related pneumonia. It also evaluated virological outcomes. Our findings could not provide any definitive conclusion. However, it is noticeable that there was no significant difference in treatment escalation as well as on secondary clinical outcomes (ventilator-free days, mortality at day 14 and day 28) between groups. In addition, there was no significant reduction in the respiratory tracts and plasma viral load between admission and day 7.

A recent study suggested that hydroxychloroquine could significantly reduce the viral load [7], but it was conducted in outpatients and a recent trial [10] performed in the ICU failed to demonstrate any benefit of lopinavir/ritonavir on clinical outcomes and viral load reduction. The effects of hydroxychloroquine are not equivocal. In another setting that is the treatment of chikungunya infection, in spite of an inhibitory effect of chloroquine on the chikungunya virus in vitro, chloroquine’s immunomodulatory effects were associated with delayed immune responses, higher levels of viral replication, and worse illness [15, 16]. While hydroxychloroquine or lopinavir/ritonavir are largely available and cheap, the potential benefit of their administration in critically ill patients has not been evaluated so far and caution is needed before considering their broad use [17]. In our retrospective analysis conducted in the ICU, whether our patients did or did not receive a specific treatment (i.e., with either antiviral or immunomodulatory activity), a similar proportion of them needed treatment escalation (within the 24 h after the admission in the ICU). Furthermore, there was a similar viral load reduction between groups. In addition, there was a similar proportion of patients with negative respiratory tract viral shedding on day 7 according to treatment allocation. In line with this, our results cannot confirm or refute the conclusion of the Surviving Sepsis Campaign guidelines which suggest against the routine use of lopinavir/ritonavir and stated that there is insufficient evidence to issue a recommendation on the use of chloroquine or hydroxychloroquine in critically ill adults with COVID-19-related pneumonia [18].

As compared to international guidelines [18], doses of hydroxychloroquine used in our patients were lower (200 mg twice a day versus a loading dose of 400 mg twice a day followed by 200 mg three times a day) which could explain the lack of beneficial effects of our strategy. Since no clear recommendations were available at the beginning of the outbreak, we conformed to our local disease control policies.

Up to date, a few studies have reported the 28-day mortality of critically ill patients with SARS-CoV-2 [10–12, 19, 20]. The larger study coming from China included 344 patients and reported a 28-day mortality of 39% [19]. The mortality rate at day 28 in our population is slightly lower (31%) but within the range of previous reports [10–12, 19]. It still remains a high mortality rate that requires efforts to improve management and to develop specific treatments. Currently, studies investigating the effects of drugs against SARS-CoV-2 on the prognosis of critically ill patients are scarce while there is a potential risk of cardiovascular adverse-drug-reactions as recently reported [21]. Therefore, further studies are needed to establish whether specific drugs have to be employed in this indication.

### Strengths and weaknesses

This opportunistic retrospective analysis allows the first comparison of hydroxychloroquine and lopinavir/ritonavir versus standard of care only ever reported in the context of SARS-CoV-2 in critically ill patients. The strength of our analysis was the efficacy evaluation combining virological data and clinical outcomes, which allows us to confront conclusions on the potential of hydroxychloroquine and lopinavir/ritonavir in our population.

Weaknesses are related to the monocentric and retrospective analysis. In addition, during the analyzed period, the management was obviously not blinded which may have influenced the clinical decision-making process. Another limitation is the comparison of three successive periods of time during which experiences and skills of caregivers may have changed. Therefore, a trend toward lower mortality and better prognosis in the treatment groups should be taken with a lot of caution. Last, our findings were obtained in a seriously ill population and may not be generalized in less severe patients and sooner after the onset of the illness.

While our analysis was unpowered to demonstrate any beneficial or harmful effects of treatments on the prognosis of critically ill patients with SARS-CoV-2-related pneumonia, our findings may be useful in the conjunction of others in the perspective of individual meta-analysis to better investigate the potential interest of such drugs. Very recently, preliminary unpublished data from the RECOVERY trial (NCT 04381936) reported no significant difference in the primary endpoint of 28-day mortality (25.7% hydroxychloroquine vs. 23.5% usual care; hazard ratio 1.11 [95% confidence interval 0.98–1.26]; *p* = 0.10) between 1542 patients randomized to hydroxychloroquine compared with 3132 patients randomized to usual care alone [22]. Nevertheless, confirmation of these data is warranted before establishing definitive answers.

## Conclusion

This retrospective observational analysis failed to demonstrate any benefit of hydroxychloroquine or lopinavir/ritonavir as compared to standard of care only on treatment escalation during the ICU stay. In addition, there was no significant difference of respiratory tracts and plasma SARS-CoV-2 load reduction between admission and day 7.

## Data Availability

The datasets analyzed during the current study are available from the corresponding author on reasonable request.

## Abbreviations

ICU: Intensive care unit
SAPS II: Simplified Acute Physiology Score II
SOFA: Sequential Organ Failure Assessment
SARS-CoV-2: Severe acute respiratory syndrome coronavirus-2
ECMO: Extra-corporeal membrane oxygenation
IQR: Interquartile range
CT: Cycle threshold
RT-PCR: Reverse transcription polymerase chain reaction
